# A deep phenotyping experience: up to date in management and diagnosis of Malan syndrome in a single center surveillance report

**DOI:** 10.1186/s13023-022-02384-9

**Published:** 2022-06-18

**Authors:** Marina Macchiaiolo, Filippo M. Panfili, Davide Vecchio, Michaela V. Gonfiantini, Fabiana Cortellessa, Cristina Caciolo, Marcella Zollino, Maria Accadia, Marco Seri, Marcello Chinali, Corrado Mammì, Marco Tartaglia, Andrea Bartuli, Paolo Alfieri, Manuela Priolo

**Affiliations:** 1 Rare Diseases and Medical Genetics Unit, University-Hospital Pediatric Department (DPUO), Bambino Gesù Children’s Hospital, IRCSS, Piazza di Sant’Onofrio, 4, 00165 Rome, Italy; 2 University of Rome Tor Vergata, Rome, Italy; 3 Academic Department of Pediatrics, Bambino Gesù Children’s Hospital, IRCCS, Rome, Italy; 4 Child and Adolescent Psychiatric Unit, Department of Neuroscience, Bambino Gesù Children’s Hospital, IRCCS, Rome, Italy; 5 Genetica Medica, Fondazione Policlinico Universitario A. Gemelli IRCCS, Rome, Italy; 6 Dipartimento Universitario Scienze della Vita e Sanità Pubblica, Sezione di Medicina Genomica, Università Cattolica del Sacro Cuore Facoltà di Medicina e Chirurgia, Rome, Italy; 7 Medical Genetics Service, Hospital “Cardinale G. Panico”, Tricase, Lecce Italy; 8 Unit of Medical Genetics, IRCCS Azienda Ospedaliero Universitaria di Bologna, Bologna, Italy; 9 Department of Pediatric Cardiology and Cardiac Surgery, Bambino Gesù Children’s Hospital IRCSS, Rome, Italy; 10 Operative Unit of Medical Genetics, Bianchi-Melacrino-Morelli Hospital, V. Melacrino, 89100 Reggio Calabria, Italy; 11 Genetics and Rare Disease Research Division, Bambino Gesù Children’s Hospital, IRCCS, Rome, Italy

**Keywords:** Malan syndrome, Sotos syndrome 2, *NFIX*, Deep phenotyping, Overgrowth

## Abstract

**Background:**

Malan syndrome (MALNS) is a recently described ultrarare syndrome lacking guidelines for diagnosis, management and monitoring of evolutive complications. Less than 90 patients are reported in the literature and limited clinical information are available to assure a proper health surveillance.

**Results:**

A multidisciplinary team with high expertise in MALNS has been launched at the “Ospedale Pediatrico Bambino Gesù”, Rome, Italy. Sixteen Italian MALNS individuals with molecular confirmed clinical diagnosis of MALNS were enrolled in the program. For all patients, 1-year surveillance in a dedicated outpatient Clinic was attained. The expert panel group enrolled 16 patients and performed a deep phenotyping analysis directed to clinically profiling the disorder and performing critical revision of previously reported individuals. Some evolutive complications were also assessed. Previously unappreciated features (e.g., high risk of bone fractures in childhood, neurological/neurovegetative symptoms, noise sensitivity and Chiari malformation type 1) requiring active surveillance were identified. A second case of neoplasm was recorded. No major cardiovascular anomalies were noticed. An accurate clinical description of 9 new MALNS cases was provided.

**Conclusions:**

Deep phenotyping has provided a more accurate characterization of the main clinical features of MALNS and allows broadening the spectrum of disease. A minimal dataset of clinical evaluations and follow-up timeline has been proposed for proper management of patients affected by this ultrarare disorder.

**Supplementary Information:**

The online version contains supplementary material available at 10.1186/s13023-022-02384-9.

## Introduction

Malan syndrome (MALNS; MIM #614753), previously called “Sotos syndrome 2”, is an ultrarare overgrowth syndrome (OGS) characterized by postnatal overgrowth, macrocephaly, a distinctive facial gestalt, skeletal defects, developmental delay (DD)/intellectual disability (ID), and behavioral anomalies [1]. MALNS is caused by haploinsufficiency of the nuclear factor I X gene (*NFIX*; MIM #164005), due to either heterozygous chromosomal microdeletions involving the 19p13.2 region or loss-of-function (LoF) variants in the *NFIX* gene, the latter almost exclusively clustering within exon 2 [1, 2]. Based on the number of known affected individuals, the prevalence of this disease is estimated as 1/1.000.000 [3]. To date, less than 90 affected individuals have been reported [2, 4–7]. Interestingly, no clinically relevant genotype–phenotype correlations were observed comparing individuals with intragenic variants and gene deletions, except for a significantly higher frequency of epilepsy in individuals carrying *NFIX* microdeletions. This finding was explained as due to a contiguous gene effect [2]. MALNS is inherited in an autosomal dominant manner and is allelic to Marshall–Smith syndrome (MSS, MIM #602535), which is characterized by postnatal failure to thrive, short stature, dysostosis, post-natal failure to thrive, typical facial gestalt, respiratory compromise, and moderate to severe DD/ID [8–10]. In MSS, *NFIX* variants (frameshift variants or intragenic deletions confined to exons 6 to 7) escape nonsense-mediated mRNA decay (NMD), leading to anomalous proteins with an aberrant shared C-terminus, with preserved DNA binding and dimerization, resulting in a possible dominant-negative effect [1, 10]. By comparing the main features of MALNS and MSS, the two conditions have been characterized as clinically distinct and allelic entities despite some clinical overlap [2]. Recently, a more severe ID, impaired speech and language, less adaptive behavior skills, and reciprocal social interaction have been reported in MSS compared to MALNS [11]. The main features of MALNS according to previous literature and differential diagnosis are summarized in Table 1.

**Table 1:** Main features of MALNS according to previous literature (Priolo et al., 2018) and its differential diagnosis

Generalized overgrowth and macrocephaly	Prenatal overgrowth: MALNS may result in large for gestational age (LGA) newborns with weight at birth > 2 SDS in 15% of cases Post-natal overgrowth: it is observed in childhood and adolescence with a height > 2 SDS reported in 56% of cases. The final height falls within two SDS from the mean in two-thirds of individuals Macrocephaly is a ubiquitous sign in generalized overgrowth syndromes. In MALNS it is observed in > 75% of individuals
Facial gestalt	Long and narrow face with a triangular shape, high and prominent forehead, short nose with anteverted nares, everted lower lip and small mouth and prognathia/prominent chin becoming more evident in adulthood. Blue sclerae are frequently observed. Other less frequent features include highly arched palate and dental crowding, sparse hair, loose and soft skin, and facial asymmetry
Peculiar neuropsychiatric behavior and intellectual disability	Both features are quite frequent/constant. Although few studies have been published [2, 11], intellectual disability seems to vary from moderate to severe. Anxiety and noise sensitivity are other neuropsychiatric features frequently reported. In some cases, self- or hetero-aggressive behavior is described. Autistic features have been observed in 31% of individuals [2]. These features impede adequate interaction between individuals and environment leading to challenging behavior [12]
Neurological features	Hypotonia is observed in 76% of cases. Seizures and electroencephalogram (EEG) anomalies are common and more frequently observed among individuals with *NFIX* microdeletions. Central nervous system anomalies characterized by wide ventricles, Corpus Callosum Hypoplasia, and Chiari malformation type 1 are more frequent than brain atrophy. Other neurological findings are different degrees of Optic Nerves Hypoplasia in 13 cases
Musculo-skeletal anomalies	Bone age is advanced in 80% of individuals. Scoliosis, hyper-kyphosis or hyper-lordosis, pectus excavatum/carinatum, slender habitus and long hands are extremely frequent
Ophthalmological features	Mainly represented by strabismus and refractive disorders such as myopia, hypermetropia and astigmatism, with an overall frequency of 76%. Other findings are nystagmus and strabismus
Cardiovascular diseases	Four individuals with aortic root dilatation and one with pulmonary artery dilatation have been reported [2, 13, 14]
Differential diagnosis	Sotos syndrome (MIM 117550), which is caused by heterozygous mutation in *NSD1* (MIM 606681) or by deletions within the 5q.35 region. Weaver syndrome (MIM 277590), which is caused by heterozygous mutation in *EZH2* (MIM 601573). Other syndromes with marfanoid habitus and ID

## Results

**Fig. 1:**
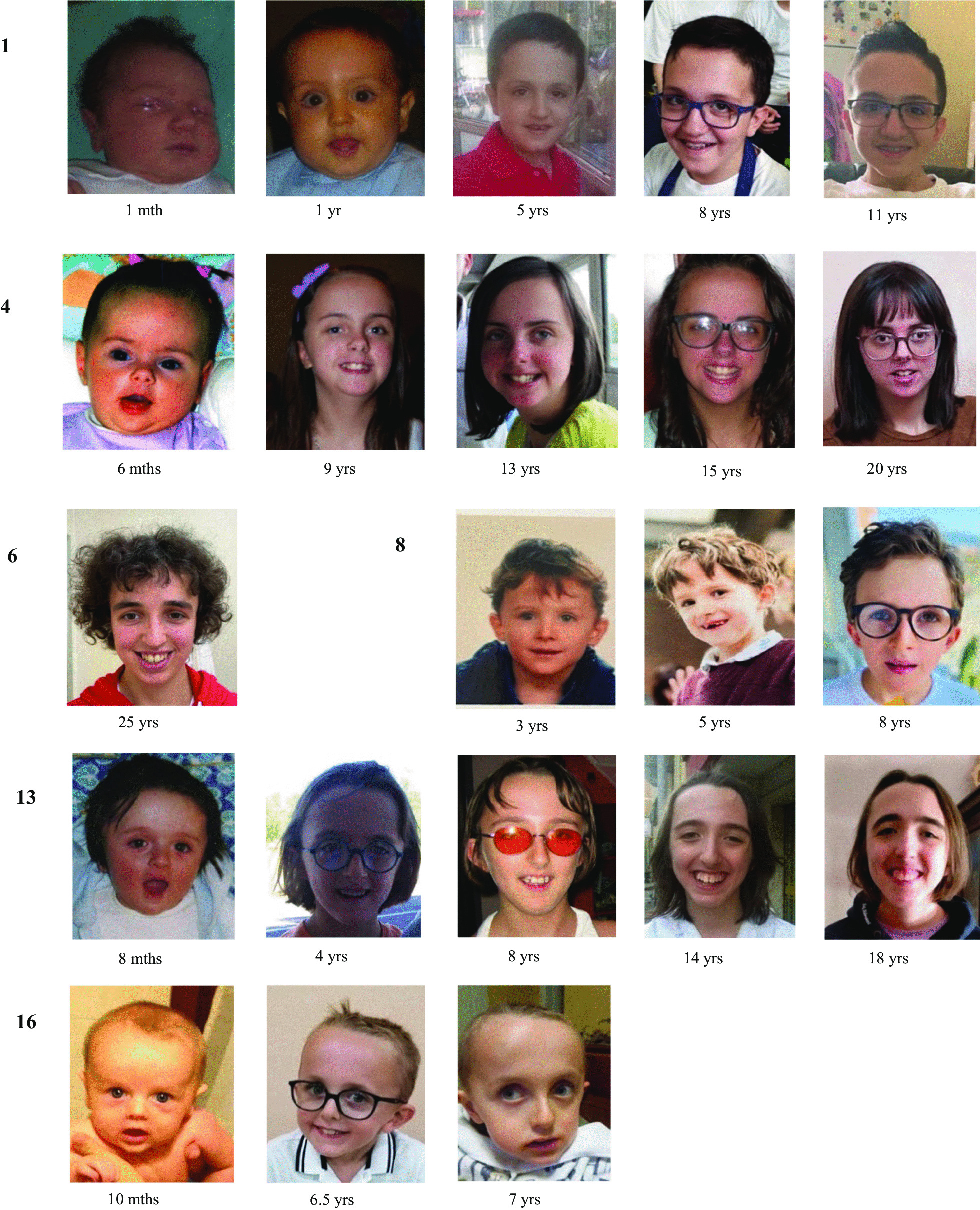
Facial feature panel of presently reported 16 individuals with Malan syndrome, with evolving facial appearance. Individuals are listed according to Table 2. Age in months (mths)/years (yrs) is reported below each picture. For detailed descriptions please see Tables and text

**Fig. 2:**
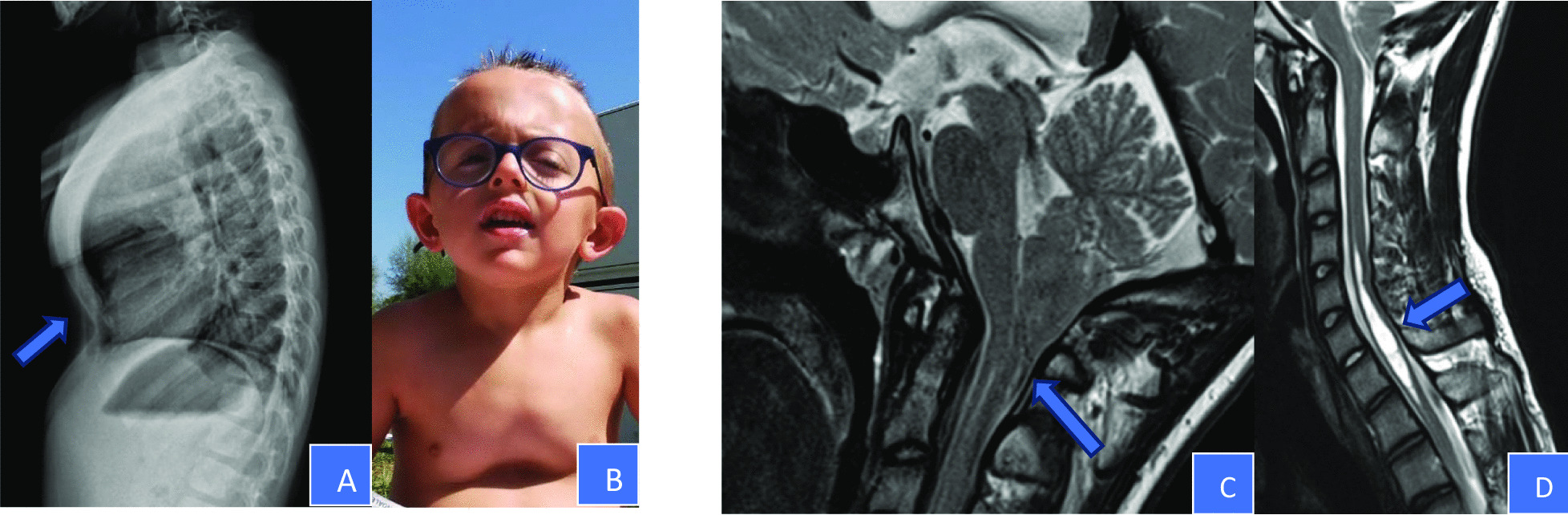
Musculoskeletal anomalies: **A** and **B** skeletal Xray and photo of case 16, that showed marked kyphosis with pectus carinatum in the upper half of the sternum and excavatum in the lower half. **C** and **D** MRI performed on case 1. The patient presented a Chiari malformation type I with a protrusion of cerebellar tonsils through the foramen magnum of 11 mm. Presence of syringomyelic cavity in the cervical spine, affecting the tract C5–C7

**Fig. 3:**
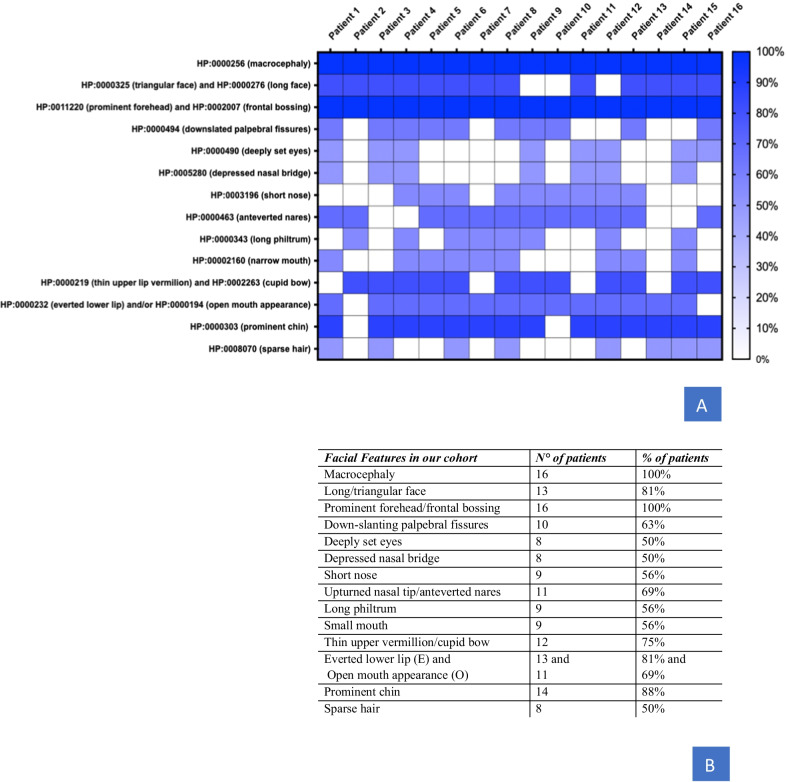
**A** Heatmap depicts common facial features for patients carrying NFIX deleterious variants by using the specific Human Phenotype Ontology (HPO) annotation (rows), which were retrieved from published studies and our cohort (columns). Phenotypic enrichment is shown according to the features’ recurrence labeled by the increment of color degree. The items with no features available were labeled white. **B** Is it possible to observe the numbers of patients presenting the specific feature (N°) and the percentage of the feature (%)

1. *General cohort assessment* Nine males and seven females, with a median age of 13.4 years (IQR range: 3–25.6 years) were enrolled. Main clinical features are reported in Figs. 1, 2 and Table 2. Detailed profiling of facial features is provided in Fig. 3. The molecular characterization of the entire cohort is provided in Table 3. Fourteen individuals had *NFIX* pathogenic SV, and two carried *NFIX* microdeletion. Seven individuals had previously been reported [2]. Nine new individuals with MALNS have been described.

**Table 2:** Main features and molecular characterization of 16 Malan syndrome patients

Patients	Priolo 2018	Present report	Present report	Priolo 2012	Gurrieri 2015	Present report	Gurrieri 2015	Present report	Gurrieri 2015	Present report	Priolo 2018	Present report	Priolo 2018	Present report	Present report	Present report	Total N = 16 (%)
	1	2	3	4	5	6	7	8	9	10	11	12	13	14	15	16	
*Main features of our cohort of Malan syndrome patients and molecular characterization*																	
Nationality	Italian	Italian	Italian	Italian	Italian	Italian	Italian	Italian	Italian	Italian	Italian	Italian	Italian	Italian	Italian	Italian	
Sex	M	M	M	F	M	F	F	M	F	F	F	M	M	F	M	M	9 M and 7 F
Age (in years)	12.5	8.2	14.6	20.2	17.2	25.6	9.9	8.4	8.5	3.7	16.1	14.2	7.1	18.5	17.2	7.4	13.4 median
**Auxologic**																	
BMI (Kg/m^2^) at last examination	18.5	13.5	15	20.5	22.5	20.5	15.8	17.0	12.0	15.9	22.3	16.5	13.2	17.4	16.0	18.6	17.5 median
**Prenatal growth**																	
Weight > 2SDS	+	−	−	+	−	−	−	−	−	−	−	−	−	−	−	−	2 (13%)
Length > 2SDS	+	−	−	+	−	−	−	+	−	−	−	−	−	−	−	+	4 (25%)
OFC > 2SDS	+	−	+	+	−	−	−	+	+	−	−	−	+	+	+	+	9 (56%)
**Postnatal growth**																	
Length > 2SDS	+	−	−	+	+	−	+	−	+	−	−	−	−	+	+	−	7 (44%)
OFC > 2SDS	+	+	+	+	+	+	+	+	+	+	+	+	+	+	+	+	16 (100%)
**Neurological**																	
Neonatal hypotonia	−	+	−	+	+	−	−	+	−	+	+	+	−	+	−	−	8 (50%)
Intellectual disability	+	++	+	+	+	+	++	++	++	DD	+	++	+	+	++	+	16 (100%)
Epilepsy	−	−	−	−	−	−	−	−	−	−	−	−	−	+	−	+	2 (13%)
Unspecific isolated EEG anomalies	+	−	+	+	+	+	−	+	−	−	+	−	−	−	+	−	8 (50%)
Behavioral anomalies*	+	+	−	+	+	+	+	+	+	+	+	+	+	+	+	+	15 (94%)
Noise sensitivity	+	+	−	+	+	+	+	+	−	+	+	+	−	+	+	+	13 (81%)
Others (EA, D, NV, PF, WG)	PF, NV	−	−	−	−	−	WG	−	−	−	−	−	−	−	EA	−	3 (19%)
**Brain MRI**																	
Wide ventricles	+	−	−	−	−	+	+	−	+	−	+	−	+	+	−	+	8 (50%)
Hypoplastic corpus callosum	−	−	−	+	−	−	+	+	+	−	−	+	−	+	+	+	8 (50%)
Brain atrophy	−	−	−	−	−	−	−	−	−	−	−	−	−	−	−	−	0 (0%)
Chiari malformation type 1	+	+	−	−	−	−	−	+	−	−	+	−	+	−	−	+	6 (38%)
**Ophthalmological**																	
Refractive disorders M, H, A	M	−	H	M	M	M, A	M	H, A	−	−	M	H	H	M, A	M	M	13 (81%)
Esotropia	+	−	+	−	−	+	+	−	−	−	+	+	+	−	+	+	9 (56%)
Strabismus	−	−	+	+	+	−	−	+	−	+	−	+	+	+	+	+	10 (63%)
Nystagmus	−	−	+	+	−	−	−	+	−	−	−	−	+	−	−	+	5 (31%)
Blue sclerae	+	−	−	+	+	+	+	−	+	+	+	−	+	+	−	+	11 (69%)
Polar cataract	+	−	−	−	+	−	−	−	−	−	−	−	−	−	−	−	2 (13%)
Optic nerve hypoplasia	−	−	−	+	+	−	+	−	+	−	−	−	−	−	−	−	4 (25%)
Optic disk pallor	+	−	−	+	−	−	+	−	+	−	−	−	−	−	−	−	4 (25%)
**Musculoskeletal**																	
Slender habitus	+	+	+	+	+	+	+	+	+	+	+	+	+	+	+	+	16 (100%)
Ligamentous hyperlaxity	−	−	−	+	+	−	−	−	−	−	−	−	−	+	−	−	3 (19%)
Long hands	+	−	−	+	+	+	+	−	+	+	−	+	−	+	+	−	10 (63%)
Hyper-kyphosis	−	−	−	−	+	−	−	−	−	−	−	−	+	−	−	+	3 (19%)
Hyper-lordosis	−	−	−	−	−	+	−	+	−	−	−	−	−	−	+	−	2 (13%)
Scoliosis	+	−	+	+	−	+	−	+	−	−	+	+	−	+	+	−	9 (56%)
Pectus excavatum/carinatum/both	±	−/−	−/+	±	−/−	±	±	−/−	±	±	−/−	−/−	±	−/−	±	+/+	8 (50%) Exc, 1 (6%) Car, 1 (6%) Mix
Pes Planus	+	+	−	+	+	+	−	+	−	+	−	+	+	+	−	+	11 (69%)
Diaphyseal bones fractures	−	−	−	+	−	+	−	+	−	−	−	−	−	+	+	−	5 (31%)
DXA	−	−	−	+	−	−	−	−	−	−	−	−	+	−	−	−	2 (13%)
**Cardiovascular**																	
Aortic root dilatation	−	−	−	−	−	−	−	−	−	−	−	−	−	−	−	−	0 (0%)
Mitral valve regurgitation	−	−	+	−	+	−	−	−	−	−	+	−	−	+	+	−	5 (31%)
**Gastrointestinal**																	
Hepatomegaly	+	−	−	−	−	−	−	−	−	−	+	−	+	−	−	+	4 (25%)
Constipation	+	+	−	−	−	−	+	+	−	+	−	+	+	−	−	+	8 (50%)
**Orodental**																	
Malocclusion	−	−	+	+	−	+	−	−	+	−	−	+	−	−	+	+	7 (44%)
Ogival palate/overcrowded teeth	+	−	−	+	+	+	−	−	−	−	−	+	+	+	+	+	9 (56%)
Caries	−	−	−	+	−	+	+	−	+	−	−	−	+	−	−	+	6 (38%)
Oral apraxia/hypersalivation	−	+	−	+	−	−	−	+	+	−	−	−	+	−	−	−	5 (31%)
**Cancer**	−	−	−	−	−	−	−	+ (WT)	−	−	−	−	−	−	−	−	1 (6%)
**ART**	+	−	−	−	−	−	−	−	−	−	−	−	−	+	−	−	2 (13%)

*A* astigmatism, *ART* assisted reproductive technology, *D* dizziness, *DD* developmental delay, *DXA* dual-energy Xray absorptiometry, *EA* episodic ataxia, *H* hypermetropia, *M* myopia, *NV* nausea or vomiting, *PF* postural fainting, *WG* wide-based gait, *WT* Wilms’s tumor

*Mainly anxiety or autistic-like behaviour. Severity of Intellectual Disability is reported as + for mild to moderate and ++ for moderate to severe

**Table 3:** Detailed molecular characterization of MALNS patients: seven patients were previously reported, and nine patients are new reports

Patients		NFIX variants	Aminoacid change	Microdeletions
1	Priolo 2018	c.[28-1G>A;28-12T>A;28- 13T>A]	p.Asp10ProfsTer5	–
2	Present report	c.95delA	p.Asn32ThrFsTer25	–
3	Present report	c.143T>A	p.Met48Lys	–
4	Priolo 2012	c.157_177del	p.Glu53_Glu59del	–
5	Gurrieri 2015	c.191delA	p.Lys64SerfsTer30	–
6	Present report	c.198dup	p.Glu67ArgfsTer60	–
7	Gurrieri 2015	c.347G>C	p.Arg116Pro	–
8	Present report	c.370_372delinsA	p.Asp124fsTer	–
9	Gurrieri 2015	c.373A>G	p.Lys125Glu	–
10	Present report	c.382C>T	p.Arg128Trp	–
11	Priolo 2018	c.499C>A	p.His167Asn	–
12	Present report	c.599_602delATAG	p.Asp200ValfsTer18	–
13	Priolo 2018	c.859_860insG	p.Glu287GlyfsTer5	–
14	Present report	c.1021del	p.His341ThrsTer52	–
15	Present report	–	–	Microdel 19p13.2—687–793 Kb (*CACNA1A* deleted)
16	Present report	–	–	Microdel 19p13.2 – 134 Kb
2. *Cardiovascular assessment* Cardiological evaluations with EcoCG were performed on all subjects and did not identify major cardiac anomalies or malformations nor dilatation of the aortic bulb or pulmonary artery. Mitral regurgitation (MR) was observed in 31% of cases. Patient 1 showed minimal pericardial effusion at EcoCG at the age of 13 years. Patient 13 showed a tricuspid aortic valve anomaly. Patient 14 presented with benign premature ventricular contractions (PVC) that were extensively investigated with a stress test and Holter ECG monitor. In this patient, mild aortic valve dysplasia was also reported.
3. *Orthopedic assessment* Musculoskeletal anomalies were frequently observed and required orthopedic evaluation and correction in some cases. Slender habitus was documented in all individuals, while long hands were observed in 63% and ligamentous hyperlaxity in 19%. Abnormal spine curvatures were observed in 75% of cases, 7 subjects had mild to severe scoliosis, 3 had hyper-kyphosis and 2 (Pts 6 and 8) with a combination of hyper-lordosis and scoliosis. One case (Pt 11) with severe scoliosis (Risser grade 3) underwent vertebral column arthrodesis at the age of 13 years old. Two additional patients (Pts 1 and 6) needed prescriptions of orthopedic insoles for the limb-length discrepancy. Patient 12 presented with severe levoscoliosis with a Cobb angle of 50 degrees, which was treated with a scoliosis brace. He is currently under orthopedic follow-up for the related high surgical risk. One case of hyper-kyphosis was surgically treated at 15 years (Pt 5). Structural deformities of the anterior thoracic wall were relatively common, with a frequency of 63%. Eight individuals showed different grades of pectus excavatum; one showed pectus carinatum, and one (Pt 16) had a severe mixed pattern of pectus carinatum and excavatum (Fig. 2A, B). The latter was investigated with an overnight pulse oximetry test which gave normal results and was evaluated by a thoracic surgeon and a pneumologist, who excluded a surgical treatment and/or noninvasive ventilation. Pes planus was recorded with high prevalence (70%). Two patients had been treated with bilateral subtalar arthrodesis (Pts 1 and 5). Diaphyseal fractures were observed in 5 subjects. Patient 4 presented with a tibia-fibula fracture at the age of 8 years. She underwent a dual-energy X-ray absorptiometry (DXA) at the age of 20 years that resulted normal. Patient 6 presented with three different episodes of tibial shaft fractures, both in right and left tibia, all occurring between 2 and 5 years, due to minor trauma. Patient 8 showed bilateral tibial shaft fractures. Patient 14 presented with unilateral tibial shaft fracture and underwent a DXA exam at the age of 15 years old that showed mild femoral osteopenia with a Z-score of − 2.1. She started supplementation with vitamin D3 and repeated the DXA at the age of 17 years that showed a rise in bone mineral density of 4.5% compared to the previous exam. Patient 15 presented with a unilateral tibial shaft fracture. Tibial fibrous cortical defect of 17 mm (Pt 1), bullet-shape epiphysis (Pt 4) and hyperostosis of the fifth finger of the hands, bilaterally (Pt 11) were also noticed.
4. *Neurological assessment* Neonatal hypotonia was observed in 50% of our cohort. The EEG showed isolated unspecific anomalies in 8 individuals (50%), 7 of them carrying an intragenic *NFIX* variant and 1 having a *NFIX* microdeletion encompassing the *CACNA1A* gene. Patient 3 carrying an intragenic *NFIX* pathogenic variant was treated for EEG anomalies alone (detected in the second year of life) with valproic acid for 1 year until the normalization of EEG pattern. Two patients presented with epilepsy (Pts 14 and 16). Patient 14 experienced an episode of seizure at the age of 8 years with a positive EEG. She started a treatment with Topiramate that was interrupted at the age of 15 years due to the normalization of the EEG pattern. Patient 16 experienced a first episode of tonic–clonic seizures with loss of consciousness at the age of 6.5 years with a positive EEG. For this reason, he started antiepileptic therapy with valproic acid. Due to the relapse of symptoms with a new critical episode, drug therapy was modified.
  All individuals were screened for brain malformation by MRI. Ventriculomegaly (50%) and different degrees of CCH (50%) were reported. CM1 was observed in 38% of cases. Patient 1 presented with a combination of CM1 and syringomyelia, a well-known association [15] (Fig. 2C, D) with a syringomyelic cavity in the cervical spine (C5-C7 segments). He performed somatosensory evoked potentials (SEPP), which resulted negative, and was evaluated by a neurosurgeon who suggested a 6-month follow-up with MRI. Patient 9 had a multicystic pineal gland and two leptomeningeal cysts at MRI; in addition, this subject showed bilateral sensorineural hearing loss previously treated with cochlear implant as a previously described sign [2]. Other observations were dysgiria and megalencephaly associated with dilatation of central canal of the spinal cord (Pt 8), punctiform area of gliosis in deep white brain matter, likely due to perinatal distress (Pt 13), septum pellucidum cysts (Pt 14) and dystopic neurohypophysis (Pt 16). Neurovegetative anomalies and gait disturbances were also noticed. Patient 1suffered from postural fainting and episodes of vomiting; patient 7 suffered from frequent falls; patient 15had bouts of dizziness, nausea, vomiting, and ataxic gait treated with acetazolamide with amelioration in symptoms.
5. *Neuropsychiatric assessment* All individuals had ID. Clinical diagnosis of mild ID was made only for one individual, while the other showed moderate or severe degree of ID. Due to young age, patient 10 received a diagnosis of developmental delay (DD). Ninety percent of individuals showed language impairment of various degrees, generally moderate or severe, in some cases with absence of speech. In general, expressive language was more impaired than receptive language. Almost all individuals presented with behavioral problems, such as anxiety and, to a lesser extent, autistic-like behaviour. Patient 6 presented with an increase in anxiety and self-aggressive behavior associated with a sleep disorder at the age of 24 years; for this reason, she started drug therapy with Mirtazapine, with amelioration in symptoms. Diminished visuomotor integration abilities were a frequent finding observed in virtually all patients. Occasionally we also suspected oculomotor apraxia in some MALNS individuals, but their neurological assessment has been separately performed and results will be provided in a separate manuscript (Alfieri et al., submitted). High sensitivity to noises was referred in 13 (81%) patients, in three cases associated with photophobia (Pts 1, 5 and 14) (not reported in Table 1). Three patients were recommended to use colored lenses (Pt 5, 14 and 16), with amelioration in photophobia and social interactions.
6. *Ophthalmological assessment* A high frequency of refractive disorders with a prevalence of 80% was observed. Four individuals had hypermetropia and nine had myopia. Three of them also presented with astigmatism. Other frequent findings were esotropia, (56%), strabismus (63%) and nystagmus (31%). Patient 11 underwent surgical correction for esotropia at 16 years of age and performed several visual evoked potentials (VEPs) and electroretinogram tests that showed altered cortical retinal transmission with normal retinal function. Anomalous VEP patterns were evidenced in patient 1, as well. Blue sclerae were observed in 69%. Bilateral polar cataracts in two cases (Pts 1 and 5) were reported. Four patients (Pts 4, 5, 7 and 9), all previously reported in the literature, presented with ONH while 4 patients (two previously reported Pts 7 and 9 and two new report Pts 1 and 4), presented with optic disk pallor.
7. *Orodental assessment* High arched palate with dental overcrowding was observed in 56% of cases. Different types of dental malocclusion (both class II and III of Angel’s classification) were observed, together with an open bite in 44%. Caries were observed in 38%. Oral apraxia and sialorrhea were noticed with an occurrence of 31%.
8. *Other relevant clinical findings* BMI and BMI-SDS were assessed in all participants resulting in a median BMI of 16.5 (interquartile (IQR): 13.2–22.5) in males and 17.4(IQR: 12–22.3) in females, with 6 individuals presenting with severe thinness (considered as BMI-SDS < 2SD or < 3rd percentile) (Additional file 1: Fig. S1). Two of them underwent nutrition assessment by an expert dietitian that confirmed normal intake of micro and macro-nutrients for age. Hepatomegaly was found in 25% of cases, a finding previously unappreciated. Constipation was a frequent observation with a prevalence of 50%. Two individuals underwent orchidopessis for bilateral cryptorchidism (Pts 2 and 3), and one (Pt 13) had surgical correction of IV grade hydronephrosis due to pyeloureteral junction stenosis associated with oligohydramnios as a previously described sign [2] and surgical correction for bilateral inguinal hernia. Patient 8 showed clitoral hypertrophy, with P1B1 Tanner stage. Frequent infections have been observed in patient 9 and patient 7, the latter also showing hypogammaglobulinemia. Patient 8 had a diagnosis of Wilms' Tumor of the left kidney with subcentimetric pulmonary metastasis at 5 years. He underwent chemotherapy and subsequently nephrectomy, according to TW2003 protocol for Wilms’ Tumor treatment developed by Associazione Italiana Ematologia e Oncologia Pediatrica (AIEOP) [16]. He is off therapy since December 2018.

## Discussion

**Fig. 4:**
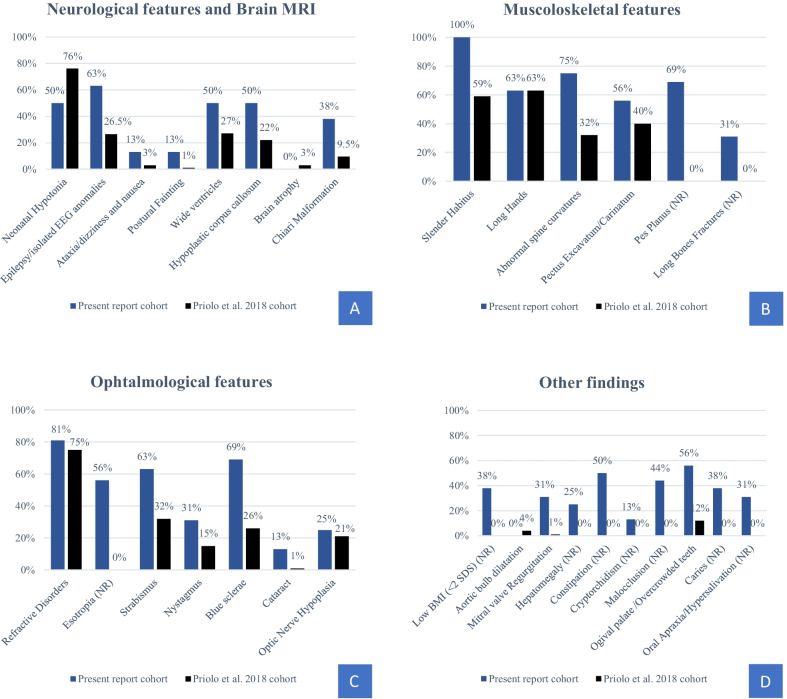
Compared frequencies of main Malan syndrome features between our cohort and Priolo et al., 2018 cohort. **A** Neurological features and Brain MRI. **B** Musculoskeletal features. **C** Ophthalmological features. **D** Other findings. Ten new features were reported among our cohort, indicated as New Report. Detailed information was reported in the text. *NR* New report

Figure 4 and Additional file 1: Table S1 report the comparison between our single-center cohort and the previously reported multicenter cohort [2]. On the whole, we found significant differences in the occurrence of many signs with respect to the previous cohort data and added ten new features to the spectrum of presentation in MALNS (Additional file 1: Table S1 shows a comparison of the most significant signs/features). These apparent discrepancies between the two series may be easily explained. Some features (i.e., altered EEG patterns identified through systematic EEG recording, neurovegetative signs, Chiari malformation, subtle corpus callosum hypoplasia, esotropia, and gastrointestinal signs) were assessed in all patients or asked to the caregivers, based on the authors’ previous experience (M.M. and M.P). Other signs (i.e., long bone fractures, and transiently reduced bone density) emerged after an accurate personal history evaluation that was possible by close periodic follow up. Musculoskeletal anomalies scored with significant differences in almost all features. Interestingly, a higher prevalence of abnormal spine curvatures was identified allowing a subclassification of spinal curvature anomalies. Pes Planus is a new but persistent observation surgically treated in two cases. Long bone fractures were evidenced in five individuals. The presence of diaphyseal bone fractures and reduced bone mineral density at DXA in one of them might be indicative of a higher risk of osteopenia/osteoporosis, which could overlap with a similar finding in MSS patients, who typically present with marked osteopenia [2]; however, the control DXA in this patient at the age of 17 was normal as the DXA performed at the age of 20 in one other of the previously fractured patients (Pt 4). Further studies are needed to investigate bone mineral density by performing DXA exams in post-pubertal MALNS individuals.

Due to the presence of slender habitus and the possible presence of a systemic score ≥ 7 of the revised Ghent criteria (e.g., scoliosis, wrist and thumb sign, pectus carinatum, myopia or mitral valve prolapse) [17]. Marfan syndrome (MIM 1547009) should be considered as a possible differential diagnosis but it can be easily ruled out by the constant presence in MALNS patients of ID and the absence of typical Marfan facial gestalt or aortic root enlargement.

MALNS may be misdiagnosed as a syndrome with marfanoid habitus and ID (e.g. homocystinuria [MIM 236200], Snyder–Robinson syndrome [MIM 309583], marfanoid mental retardation syndrome [MIM 248770], Lujan–Fryns syndrome [MIM 309520]), though the facial gestalt and clinical presentation of these disorders significantly differ from MALNS. Molecular confirmation is required to assess proper diagnosis.

Epilepsy and isolated EEG anomalies were also more frequently observed. Individuals with *NFIX* intragenic variants were prone to develop aspecific EEG anomalies, which did not require anti-epileptic therapy. Brain MRI was performed in all individuals documenting a higher prevalence of structural brain anomalies (wide ventricles, CCH and CM1) confronted with the previous series. Recurring neurovegetative signs also emerged as a relevant clinical issue in MALNS. The previous series reported 1 patient with postural fainting. By retrospectively reviewing all other MALNS individuals affected with 19p13.2 microdeletions involving *NFIX*, three additional cases with cyclic vomiting, dizziness, nausea, and sporadic ataxic gait were recognized [18, 19]. It has been hypothesized that co-occurring *CACNA1A* deletion could be an associated cause in the pathogenesis of these signs [19]. Neurovegetative signs and gait disturbances have also been occasionally described in CM1 as brainstem/cerebellar-related symptoms [20, 21]. By identifying the presence of CM1 in some of the 7 MALNS individuals presenting with neurovegetative symptoms, we speculate that mixed mechanisms likely trigger them. Three out seven patients had a co-occurring *CACNA1A* deletion, one subject had a microdeletion not involving *CACNA1A* but was affected with CM1 and syringomyelia and one subject (Pt 1) carrying a pathogenetic *NFIX* variant had CM1 with syringomyelia (Additional file 1: Table S2). Two other cases had neither *CACNA1A* deletion nor CM1. We concluded that for the first one, previously described [2], postural hypotension was a too general sign to be considered a true neurovegetative one. Patient 7 in the present series showed unsteady gait and frequent falls, but never showed neither neurovegetative symptoms such as vomit, nausea, or abdominal pain nor neurologic symptoms such as dizziness or vertigo. So, we can hypothesize that her clinical picture could be explained by the frequent finding of diminished visuomotor integration abilities within the MALNS population.

Neuropsychiatric and behavior evaluation revealed moderate to severe ID in almost all patients with the exception of one who was diagnosed with mild DD (Pt10). Behavioural problems with peculiar anxious profile have been evidenced in more than 50%. Diminished visuomotor integration abilities seem to be a neuropsychiatric hallmark in MALNS such as noise sensitivity and photophobia. Colored lenses are currently used by autistic patients to reduce light stimuli triggers and improves social tasks [22]. Three patients of the present series (Pt 5, 14 and 16) successfully adopted the same strategy with the benefice of reducing anxiety outbursts. Due to the complexity of the methodology of assessment, neuropsychiatric results will be provided in a separate manuscript (Alfieri et al., submitted).

A multicystic pineal gland and meningeal cysts have also been recorded. Both signs have been previously recorded in MALNS [2, 18] although these are quite common MRI finding in general population [23, 24]. Nonetheless, their progression should be monitored to prevent associated neurologic symptoms.

A comparable frequency of refractive disorders with respect to previous reports has been observed. On the other hand, a higher frequency of strabismus and nystagmus has been recorded. Of note, esotropia had a prevalence of 56%; bilateral polar cataract (with a probable congenital etiology) was detected in patients 1 and 5 during the current evaluation although they were previously described [2]. Optic Nerve Hypoplasia (ONH) and Optic Disk Pallor (ODP) were evidenced in 25% of cases, respectively.

Cardiovascular diseases had been previously claimed as a possible main feature of MALNS due to the frequent observation of Marfanoid habitus [2]. None of the individuals in the present series showed aortic bulb or pulmonary artery dilatation. Minor anomalies such as low-grade mitral regurgitation (MR) in 31% have been observed. In a large cohort study on the adult population (The Framingham Heart Study) MR was detectable at echocardiographic examinations in a vast control population, resulting in trace or mild MR [25]. We might conclude that these minor anomalies are not sufficient to classify MALNS as a condition predisposing to cardiovascular disease and/or heart anomalies.

Visceromegaly is a well-known association in OGS [26]. Isolated hepatomegaly was observed in 25% of MALNS patients here described. Different degrees of constipation were observed in 50% of our cohort, in some cases requiring pharmacological therapy with polyethylene glycol. Functional constipation is a frequent problem in patients with ID or DD [27]. Nonetheless, due to the high occurrence in MALNS population and the consequent impact on the quality of life, great attention should be paid to this complication.

Six individuals with a BMI-SDS < 2SDS have been observed, indicative of severe thinness (Additional file 1: Fig. S1). Interestingly, *NFIX*-null mice are characterized by a reduced body size and inability to fully extend their limbs, suggesting a possible muscular phenotype [28]. *NFIX* drives transcriptional changes from embryonic to fetal myogenesis by specifically activating fetal genes [29]. Mice lacking *NFIX* show reduced myofiber cross sectional area. NFIX also acts through an inhibitory mechanism at the promoter of the gene that encodes for *myostatin,* a TGF- β family member with anti-myogenic properties [30]. This finding is consistent with the hypothesis that MALNS individuals could show a reduced muscular mass with the final consequence of the inability to gain weight, despite adequate nutrient intakes. The Marfanoid-progeroid-lipodystrophy syndrome (MPLS) (MIM 616914), a newly recognized fibrillinopathy caused by pathogenic dominant negative variants clustering in *FBN1* exon 65 [31], is also characterized by extremely low BMI with reduced subcutaneous fat. Aberrant activation of the TGF-β signaling pathway in a SMAD-dependent manner has been demonstrated in a subset of individuals affected with MPLS. The TGF-β plays a role in fat metabolism with an inhibitory action on human adipose tissue development [32]. This evidence suggests that MALNS individuals may present with a reduced BMI caused by either muscular or fat involvement likely through the same TGF-β signaling pathway dysregulation. Further studies are needed to confirm the role of NFIX LoF in TGF-β signaling regulation.

A higher frequency of high arched palate with overcrowded teeth has been observed, as well. Accurate orodental evaluation at diagnosis and then strict follow up is highly recommended.

The second case of cancer in MALNS was recorded (Pt 8). OGS are usually associated with an increased risk of tumors with only slightly increased prevalence in most OGS when compared with the general population [33]. Wilms tumor is an embryonic tumor quite represented among OGS although with significant different prevalence in risk [33]. To date, 2 individuals (Pt 8 of the present report and Pt 43 from [2]) with MALNS with different types of neoplasms have been reported in the literature with and overall prevalence of 2.2% thus including MALNS in the same low risk group as SS and WVS with a low likelihood of developing cancer [33, 34]. We are aware that MALNS cohort is too small to calculate an absolute risk so we might suggest that routine surveillance is not recommended considering psychosocial implications for the affected individuals and their families, as well as the cost of an active instrumental cancer surveillance.

As additional information we reported two MALNS individuals conceived via Assisted Reproductive Technology (ART), one previously described (Pt 1) and one new report (Pt 14), and they add to another one previously described (Pt 21 from [2]).

### A proposal for diagnosis, management and follow up of the main complication in Malan syndrome

Due to the recent description of MALNS and its rarity, there are no current guidelines to help clinicians in diagnosis, management and follow up. Here we propose a minimal dataset of clinical evaluation to be applied to MALNS individuals to precociously identify possible complications and assure proper management and follow up (Table 4), based on previous reports and the results of our experience. We strongly suggest auxologic (with periodic BMI evaluations), orthopedic, ophthalmologic, and neurological evaluations and strict follow up. Caregivers should be properly trained to recognize subtle neurological signs (seizures and/or neurovegetative signs) and should be informed of higher recurrence of pathologic fractures of long bones in their children. DXA assessment and vitamin D dosage may be recommended during puberty to eventually provide adequate supplementation. Neuropsychiatric and behavior assessments need a separate dataset and recommendations due to their detailed phenotyping (Alfieri et al. in preparation).

**Table 4:** Proposal for a minimal dataset for clinical evaluation, management, and follow-up on Malan syndrome

Intervention	Evaluation	Frequency and follow-up
Auxological evaluation	Evaluate habitus, measurement of weight, length/height, head circumference	Complete evaluation with measurement of weight, length/height, head circumference at diagnosis. Repeat auxological evaluation every six months during the first 2 years of life, then at least annually
	Calculate BMI-SDS and calories intake, reassure about appropriateness in food intake. Variate diet. If < 2SDS evaluate calories intake	Refer to pediatrician for BMI-SDS surveillance (first evaluation after 2 years of age)
Orthopedic evaluation	Evaluate spine curvature, body length discrepancies, flat feet. When required involve specialized practitioners (e.g., orthopedic surgeon, physiatrist, physiotherapist). Perform skeletal Xray if needed	At diagnosis, then evaluate annually until puberty
	Accurate anamnesis for bone fractures. Consider DXA	DXA should be performed during puberty or earlier if presence of pathologic fractures
Ophthalmologic evaluation	Perform Ophthalmologic/Orthoptic evaluation. Search for refractive errors, nystagmus, strabismus, polar cataract, and optic disk pallor. Consider VEP and ERG to assess conduction in visual pathway	At diagnosis, then annually until puberty
Neurological evaluation I.CNS anomalies	MRI at diagnosis with critical search for CCH, wide ventricles, brain atrophy and CM1. Consider specific evaluation of optic nerve at MRI to detect Optic Nerve hypoplasia	At diagnosis. If CM1 is detected at MRI perform imaging follow-up. If CM1 is associated to syringomyelia consider neurosurgeon evaluation for proper timing of the imaging
II.Neurovegetative symptoms	Symptoms may be subtle or aspecific (e.g., vomiting/nausea, dizziness fainting). Look for CM1 and consider neurologic evaluation. Exclude other causes (e.g., cardiologic/otolaryngology evaluation)	At diagnosis, evaluate possible appropriate drug therapy, subsequent follow up
III.Epilepsy and EEG anomalies	Perform EEG in asymptomatic patients, educate caregivers to promptly recognize subtle symptoms	At diagnosis. If EEG aspecific anomalies alone are detected: watch and wait strategy, especially in NFIX SV. In patients with NFIX microdeletions consider closer follow-up due to the higher risk in developing seizures
	If onset of seizures: Perform neurological evaluation and EEG, if required start antiepileptic therapy	At diagnosis, then on neurological indication
Neuropsychiatric and Behavior evaluation	Perform neuropsychiatric and behavior evaluation Verify visuomotor abilities, noise sensitivity and photophobia. Suggest symptomatic aids (i.e., coloured glasses, recommend low voice tone) when appropriate to reduce anxiety outbursts	At diagnosis, then on neuropsychiatric indication
Orodental Odontoiatric evaluation	Evaluate ogival palate, tooth overgrowth, malocclusions, caries. Educate to proper dental care	At diagnosis, then annually or on specific indications
Gastrointestinal evaluation	Look for constipation and treat it. Perform abdominal ultrasound to look for hepatomegaly and/or kidney enlargement	At diagnosis. Refer to pediatrician for possible therapy of constipation
Neoplasm surveillance	No consistent evidence for strict neoplasm surveillance	None
Cardiological evaluation	Perform echocardiogram. Look for AD, MR, other CHD	At diagnosis. Refer to pediatrician for periodic clinical evaluation
Genetic counselling	Appropriate genomic/genetic testing to exclude parental mosaicism. Consider parental or gonadal mosaicism	At diagnosis. Prenatal counselling in families with a known MALNS offspring conceived by ART

*AD* aortic dilatation, *ART* assisted reproductive technology, *BMI-SDS* Body Mass Index-Standard Deviation Score, *CM1* Chiari malformation type 1, *CNS* central nervous system, *CHD* congenital heart disease, *DXA* dual energy absorptiometry, *EEG* electroencephalogram, *ERG* electroretinogram, *MR* mitral regurgitation, *MRI* magnetic resonance imaging, *VEP* visual evoked potentials

MALNS is inherited as an autosomal dominant condition so each individual with MALNS has a 50% chance of transmitting the disorder. To the best of our knowledge, reproductive fitness in MALNS is extremely low, due to the severe ID. Currently, no MALNS offspring have been reported in the literature. Most individuals diagnosed with MALNS carry a de novo genetic alteration (either microdeletion or SV). However, 6 previous individuals from 3 unrelated families with recurrence of disease due to either gonadal or parental mosaicism have been described [2, 6, 7]. Recommendations for the evaluation of parents of an individual with MALNS include appropriate genomic/genetic testing to exclude parental mosaicism. If the genetic alteration cannot be detected in either parent, it’s highly probable that the proband has a de novo event. However, gonadal mosaicism cannot be excluded. In that case, prenatal genetic counselling should be performed to inform parents about their residual low recurrence risk, and prenatal diagnosis could be offered. Due to the reporting of three MALNS individuals conceived by ART and with an apparently de novo pathogenic alteration, great attention should be paid in these families if other fetuses conceived by ART present as LGA.

## Conclusions

Deep phenotyping in MALNS has helped to identify a more accurate occurrence of main features and has allowed us to expand the spectrum of signs and symptoms characterizing this ultrarare disorder. The high expertise of the team involved in all the evaluations contributed to promptly identify main and critical features to eventually ensure uniformity in the treatment of complications. A minimal dataset of clinical evaluations and follow-up timeline has been proposed for proper management in MALNS. A more detailed study on behavioral disturbances is in preparation. Further studies are needed to confirm our findings in a larger cohort of MALNS individuals.

## Materials and methods

### Population and molecular diagnosis

Sixteen genetically confirmed Italian MALNS individuals were recruited from the outpatient clinic of the “Clinical Genetic and Rare Diseases” Unit, Ospedale Pediatrico Bambino Gesù (Rome, Italy), from September 2020 to November 2021 and enrolled in the study. Molecular diagnosis was attained by clinical exome sequencing (CES) or comparative genomic hybridization/SNP array, which identified intragenic *NFIX* pathogenic variants or *NFIX* microdeletions in the frame of diagnostic testing.

### Study design

All the participants underwent a multidisciplinary assessment based on the previous description of main features and complications, as previously reported [2]. They were firstly evaluated by an expert panel group (MM, MP, PA, MC), then monitored with a multistep assessment involving several specialists (e.g., neurologists, neurosurgeons, ophthalmologists, orthopedic surgeons), depending on the specific individual clinical history or needs. They underwent: (I) a thorough pediatric evaluation (MM) with the co-presence of an experienced clinical geneticist (MP); (II) a neuropsychiatric evaluation to assess cognitive, neuropsychological and psychopathological comorbidities profile characteristics (PA and CC); (III) a cardiological evaluation (MC) with echocardiogram to exclude possible heart malformation and/or aortic root or pulmonary artery dilatations. Depending on the clinical conditions of each subject, orthopedic evaluation was performed by radiological imaging to investigate skeletal anomalies (e.g., scoliosis, kyphosis/lordosis, and pectus excavatum), and neurological/neurosurgical evaluation, with CNS imaging directed to detect possible brain anomalies (e.g., Chiari malformation type 1 (CM1), wide ventricles, and corpus callosum hypoplasia (CCH).

Body mass index (BMI) and BMI-standard deviation score (SDS) were calculated according to the World Health Organization (WHO) guidelines [35] while BMI charts for boys and girls were drafted in accordance with Centers for Disease Control and Prevention (CDC) [36].

### Ethics

All procedures were in accordance with the ethical standards of the institutional and/or national research committee and the 1964 Helsinki declaration and its later amendments or comparable ethical standards. All information has been collected during the clinical assessment in Bambino Gesù Children’s Hospital, after obtainment of informed consent and registered anonymously in the electronic medical record.

## Supplementary Information

**Additional file 1.** Supplementary figures and tables.

## Data Availability

All data on our cohort were collected during medical assessment in Bambino Gesù Children’s Hospital, after obtainment of informed consent.

## Abbreviations

BMI: Body Mass Index
CCH: Corpus callosum hypoplasia
CM1: Chiari malformation type 1
DD: Developmental delay
DXA: Dual-energy X-ray absorptiometry
EcoCG: Echocardiogram
EEG: Electroencephalogram
ID: Intellectual disability
IQR: Interquartile
LGA: Large for gestational age
LoF: Loss-of-function
MALNS: Malan syndrome
MSS: Marshall–Smith syndrome
MR: Mitral regurgitation
MRI: Magnetic resonance imaging
*NFIX*: Nuclear factor I X
OGS: Overgrowth syndromes
PVC: Premature ventricular contractions
SDS: Standard deviations score
SEPP: Somatosensory evoked potentials
SS: Sotos syndrome
SV: Single variant
TGF-β: Transforming growth factor-β
US: UltraSound
URD: Ultra rare disease
WHO: World Health Organization
WVS: Weaver syndrome
