# Supplementary material for: A deep phenotyping experience: up to date in management and diagnosis of Malan syndrome in a single center surveillance report
Source: Orphanet J Rare Dis. 2022 Jun 18;17:235. doi: 10.1186/s13023-022-02384-9 (PMC9206304; doi:10.1186/s13023-022-02384-9)
Supplement: Supplementary file 1 — Additional file 1. Supplementary figures and tables. [file 13023_2022_2384_MOESM1_ESM.docx]

Supplemental Data File

Macchiaiolo M et al.

Supplemental Figures

*Supplemental Figure 1*

Females

Males


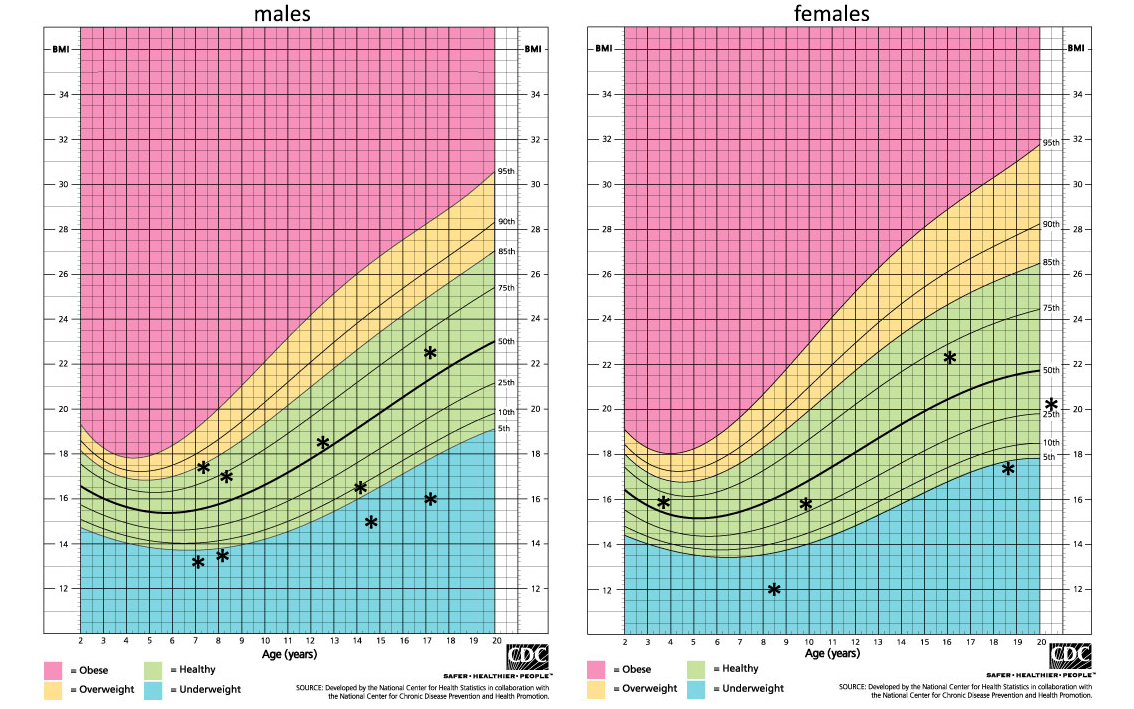

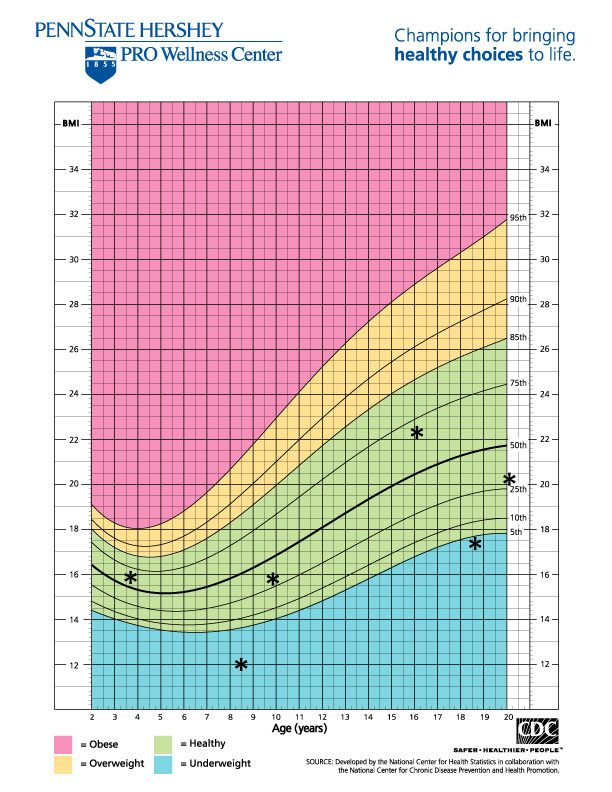


**Supp. Figure 1 Malan Syndrome BMI percentile charts:**  BMI percentile pediatric charts for males (on the left) and females (on the right). Four males and 2 females MALNS patients are below the 3^rd^ percentile for BMI. This condition is indicative for severe thinness. No overweight/obese or overweight patients (BMI>95^th^ percentile) were observed. Patient 6 is not included in BMI charts due to her age (>20 years old); her BMI was within normal range (20.6 Kg/m^2^).

Supplemental Tables

*Supplemental Table 1*

| ***Comparison of main features in Malan Syndrome between our cohort and Priolo et al. 2018 cohort*** | | | | | | |
| --- | --- | --- | --- | --- | --- | --- |
|  | | *Our cohort*  *Patients (N=16)* | | *Priolo et al. 2018*  *patients* | | *Frequency of reported data among our cohort* |
|  |  | *N* | *%* | *N* | *%* |  |
| Auxological | Low BMI (<2 SDS) | *6* | *38%* | *-* | *-* | *New Report* |
| Neurological | Neonatal Hypotonia | *8* | *50%* | *57/75* | *76%* | *Lower* |
|  | Epilepsy/EEG anomalies | *10* | *63%* | *21/79* | *26.5%* | *Higher* |
|  | Episodic Ataxia/dizziness and nausea | *2* | *13%* | *2/80* | *3%* | *Higher* |
|  | Postural Fainting | *2* | *13%* | *1/80* | *1%* | *Higher* |
| Brain MRI | Wide ventricles | *8* | *50%* | *17/63* | *27%* | *Higher* |
|  | Hypoplastic corpus callosum | *8* | *50%* | *14/63* | *22%* | *Higher* |
|  | Brain atrophy | *0* | *0%* | *2/63* | *3%* | *None* |
|  | Chiari Malformation type 1 | *6* | *38%* | *6/63* | *9.5%* | *Higher* |
| Ophthalmological | Refractive Disorders | *13* | *81%* | *60/80* | *75%* | *Higher* |
|  | Esotropia | *9* | *56%* | *-* | *-* | *New report* |
|  | Strabismus | *10* | *63%* | *26/80* | *32%* | *Higher* |
|  | Nystagmus | *5* | *31%* | *12/78* | *15%* | *Higher* |
|  | Blue sclerae | *11* | *69%* | *19/66* | *26%* | *Higher* |
|  | Cataract | *2* | *13%* | *1/80* | *1%* | *Higher* |
|  | Optic Nerve Hypoplasia | *4* | *25%* | *13/63* | *21%* | *Comparable* |
| Musculoskeletal | Slender Habitus | *16* | *100%* | *46/78* | *59%* | *Higher* |
|  | Long Hands | *10* | *63%* | *50/78* | *63%* | *Comparable* |
|  | Abnormal spine curvatures (HK, HL, S or mixed) | *12* | *75%* | *23/72* | *32%* | *Higher* |
|  | Pectus Excavatum/Carinatum (or mixed) | *10* | *63%* | *35/70* | *40%* | *Higher* |
|  | Pes Planus | *11* | *69%* | *-* | *-* | *New report* |
|  | Long Bones Fractures | *5* | *31%* | *-* | *-* | *New report* |
| Cardiovascular | Aortic bulb dilatation | *0* | *0%* | *3/79* | *4%* | *Lower* |
|  | Mitral valve Regurgitation | *5* | *31%* | *1/79* | *1%* | *Higher* |
| Gastrointestinal | Hepatomegaly | *4* | *25%* | *-* | *-* | *New report* |
|  | Constipation | *8* | *50%* | *-* | *-* | *New report* |
| Genitourinary | Cryptorchidism | *2* | *13%* | *-* | *-* | *New report* |
| Orodental | Malocclusion | *7* | *44%* | *-* | *-* | *New report* |
|  | Ogival palate /Overcrowded teeth | *9* | *56%* | *5/43* | *12%* | *Higher* |
|  | Caries | *6* | *38%* | *-* | *-* | *New report* |
|  | Oral Apraxia/Hypersalivation | *5* | *31%* | *-* | *-* | *New report* |

**Supp. Table 1 Comparison of main features frequencies in Malan Syndrome between our cohort and Priolo et al. 2018:** Frequencies of main MALNS features in our cohort vs Priolo et al., 2018 cohort. Abbreviations: HK: Hyper-Kyphosis, HL: Hyper-Lordosis, S: Scoliosis

*Supplemental Table 2*

| **Neurovegetative signs in Malan Syndrome** | | | | | | | | **Tot** |
| --- | --- | --- | --- | --- | --- | --- | --- | --- |
|  | ***Pt 1***  ***Present report*** | ***Pt 6***  ***Gurrieri 2015*** | ***Pt 38 Priolo 2018*** | ***Pt 2 Dolan 2010*** | ***Pt 4 Dolan 2010*** | ***Pt 4 Klaassens 2015*** | ***Pt 15***  ***Present report*** | ***7*** |
| *NFIX* Deletion involving *CACNA1A* | - | - | - | + | - | + | + | 3 |
| *NFIX SV* | + | + | + | - | - | - | - | 3 |
| CM1/Syringomyelia | + | - | - | - | + | - | - | 2 |
| Molecular Characterization | c.[28-1G > A;28-12T > A;28- 13T>A] p. Asp10ProfsTer5 | c. 347G>C p. Arg116Pro | c.779C > G p.Thr260Ser | Microdel 19p13.13-p13.2  Size: 1.3 Mb | Microdel 19p13.13-p13.2  Size: 0.7 Mb  (Not involving *CACNA1A*) | Microdel 19p13.2  Size: 560–640kb | Microdel  19p13.2 -  Size: 687-793 Kb |  |

**Suppl. Table 2 Neurovegetative signs in Malan Syndrome:** Patients with neurovegetative signs among our cohort and in previous literature. Abbreviations: CM1: Chiari Malformation type 1, SV: Single Variant.
